# Correction: Rame Hau et al. The Prevalence and Risk Factors Associated with the Presence of Antibiotic Residues in Milk from Peri-Urban Dairy Cattle Farms in Kathmandu, Nepal. *Antibiotics* 2025, *14*, 98

**DOI:** 10.3390/antibiotics14050473

**Published:** 2025-05-07

**Authors:** Erda E. Rame Hau, Minu Sharma, Bal K. Sharma Khanal, Peter D. Sly, Deirdre Mikkelsen, Nicholas Clark, Ricardo J. Soares Magalhães

**Affiliations:** 1 Queensland Alliance for One Health Sciences, School of Veterinary Science, The University of Queensland, Gatton, QLD 4343, Australia; 2 National Zoonoses and Food Hygiene Research Centre (NZFHRC), Kathmandu, Nepal; 3 Ministry of Agriculture and Livestock Development, Kathmandu, Nepal; 4 Children’s Health and Environment Program, Children Health Research Centre, The University of Queensland, Brisbane, QLD 4000, Australia; 5 School of Agriculture and Food Sustainability, The University of Queensland, Brisbane, QLD 4072, Australia

## 1. Text Corrections

There were errors in the original publication [1].

1. There was an error in the last sentence of the fifth paragraph of Section 1 describing “PERIMILK Study Nepal refers to a research initiative that focuses on investigating the production, distribution, and safety of milk in peri-urban areas around Kathmandu, Nepal”. A correction has been made by deleting this sentence.
2. The term “Perimilk” was included in the text of Section 1, 2.1 and the title of Supplemenatry File S1 by mistake. A correction has been made by removing the term “Perimilk” from the text. The title of File S1 has been corrected to “Risk Factors Data of Antibiotic Residues Milk Samples Nepal”.
3. The inclusion of the following last sentence in Section 4.1 was made in error: “This study was carried out after obtaining Animal Ethics Approval from the Public Health Foundation of India, the Government of National Capital Territory of Delhi (Certificate Number: IN-DL62225746874638Q)”. A correction has been made by deleting this sentence.
4. Institutional Review Board Statement: This study was carried out after obtaining Animal Ethics Approval from the Public Health Foundation of India, the Government of National Capital Territory of Delhi (Certificate Number: IN-DL62225746874638Q). The following correction has been made to the institutional review board statement: This study was carried out without the need to obtain formal ethical approval, as the investigations were part of routine disease investigations carried out by the Nepalese National Zoonoses and Food Hygiene Research Centre, Kathmandu.
5. There was an error in the Acknowledgments Section; the name “Manish Kakkar” was added by mistake. A correction has been made by removing it, and the revised acknowledgement statement is as follows: We would like to acknowledge the contribution of the farmers who participated in the study and the Ministry of Agriculture of Nepal staff who supported the fieldwork.

## 2. Errors in Figures/Tables

In the original publication [1], the term “Perimilk” was mistakenly added in Figures 1, 4 and 5, Tables 1, 2 and 4. “Perimilk” has been deleted from the top titles of Figures 1, 4 and 5, and deleted from the captions of Tables 1, 2 and 4.

1 The top title of Figure 1 has been deleted. The caption of Figure 1 has been corrected to the following: “The seven districts, including Balaju, Budhanilkantha, Chandragiri, Godawari, Kirtipur, Ramkot, and Sundarijal, involved in the study in Kathmandu, Nepal”.
2 The top title of Figure 4 has been corrected to “Total Antibiotic Residues in Nepal Study”.
3 The top title of Figure 5 has been corrected to “Ciprofloxacin Residues”, “Enrofloxacin Residues”, “Sulfamethazine Residues “, and “Sulfamethoxazole Residues”.
4 The caption of Table 1 has been corrected to “Table 1. The risk factors associated with the number of antibiotic residues (ARs) present in samples.”
5 The caption of Table 2 has been corrected to “Table 2. Risk factors associated with number of antibiotic residues exceeding maximum residue limit (MRL) in samples (no AM residue exceeding MRL (0), 1 AM residue exceeding MRL (1), and 2 or more AM residues exceeding MRL (2)).”
6 The caption of Table 4 has been corrected to “Table 4. Counts of farms, cattle, and sampled farms for each peri-urban district of Kathmandu, Nepal, included in this study.”

The authors state that the scientific conclusions are unaffected. This correction was approved by the Academic Editor. The original publication has also been updated.

## Figures and Tables

**Figure 1:**
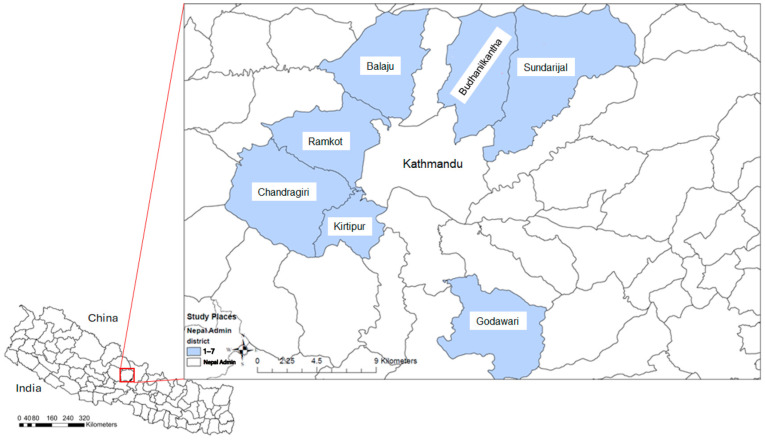
The seven districts, including Balaju, Budhanilkantha, Chandragiri, Godawari, Kirtipur, Ramkot, and Sundarijal, involved in the study in Kathmandu, Nepal.

**Figure 4:**
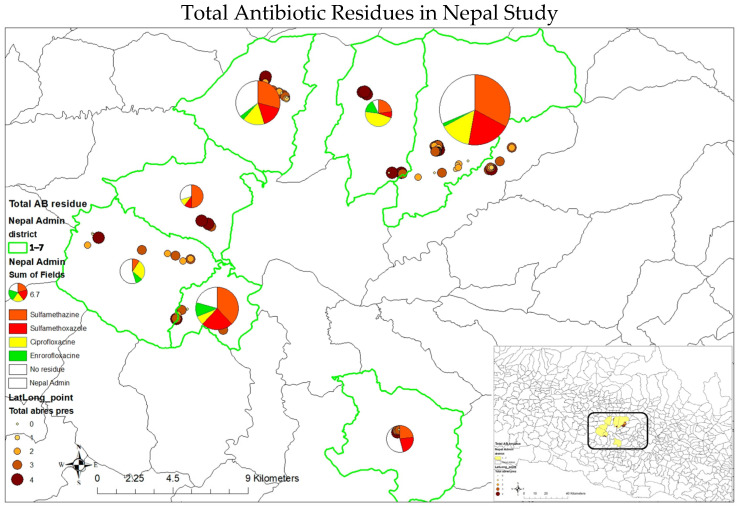
The presence of antibiotic residues in the seven peri-urban districts of Kathmandu, Nepal.

**Figure 5:**
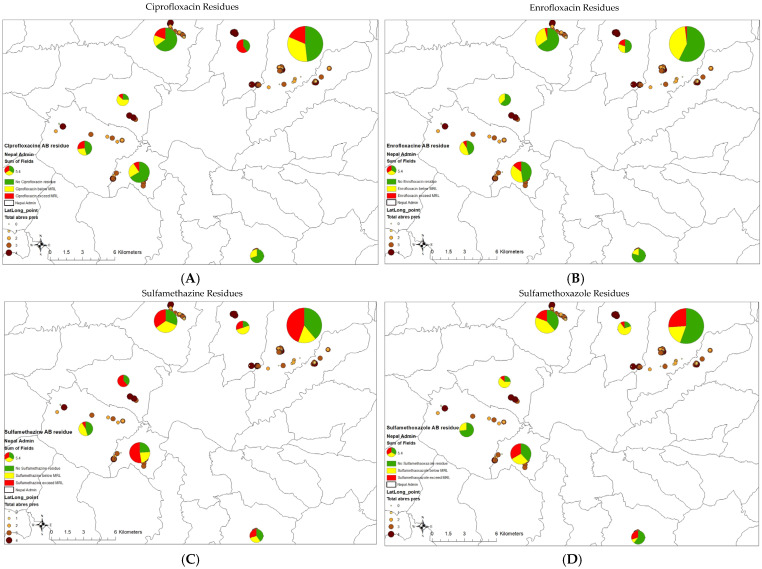
The residue profiles for the antibiotics ciprofloxacin (**A**), enrofloxacin (**B**), sulfamethazine (**C**), and sulfamethoxazole (**D**), indicating the number of antibiotic residues (AB) exceeding the maximum residual limit (MRL) compared to AB presence recorded for the seven peri-urban districts in Kathmandu, Nepal.
