# Supplementary material for: The Prevalence and Risk Factors Associated with the Presence of Antibiotic Residues in Milk from Peri-Urban Dairy Cattle Farms in Kathmandu, Nepal
Source: Antibiotics (Basel). 2025 Jan 16;14(1):98. doi: 10.3390/antibiotics14010098 (PMC11760848; doi:10.3390/antibiotics14010098)
Supplement: Supplementary file 1 [file antibiotics-14-00098-s001.zip › antibiotics-3407811-supplementary.pdf]

File S1. Risk Factors Data of Antibiotic Residues Milk Samples Nepal

Samples	Districts	AR Presence	Total AR Presence	Above MRL/Not	Total AR above MRL	Enrofloxacin Presence	Enrofloxacin above MRL	Ciprofloxacin Presence
K0101	Sundarijal	Abpresent	2abr	Yes	2mrlab	No	No	No
K0102	Sundarijal	Abpresent	2abr	Yes	1mrlab	No	No	No
K0103	Sundarijal	Abpresent	3abr	Yes	2mrlab	No	No	Yes
K0104	Sundarijal	Abpresent	3abr	Yes	1mrlab	Yes	Yes	No
K0105	Sundarijal	Abpresent	4abr	Yes	2mrlab	Yes	No	Yes
K0106	Sundarijal	Ababsent	1abr	No	0mrlab	Yes	No	No
K0107	Sundarijal	Ababsent	3abr	No	0mrlab	Yes	No	Yes
K0108	Sundarijal	Abpresent	2abr	Yes	1mrlab	No	No	No
K0109	Sundarijal	Ababsent	1abr	No	0mrlab	No	No	No
K0110	Sundarijal	Abpresent	3abr	Yes	2mrlab	Yes	No	Yes
K0111	Sundarijal	Ababsent	3abr	No	0mrlab	Yes	No	Yes
K0112	Sundarijal	Abpresent	1abr	Yes	1mrlab	No	No	No
K0113	Sundarijal	Abpresent	2abr	Yes	1mrlab	No	No	No
K0114	Sundarijal	Ababsent	0abr	No	0mrlab	No	No	No
K0115	Sundarijal	Abpresent	1abr	Yes	1mrlab	No	No	Yes
K0116	Sundarijal	Abpresent	2abr	Yes	1mrlab	No	No	No
K0117	Sundarijal	Abpresent	4abr	Yes	2mrlab	Yes	No	Yes
K0118	Sundarijal	Abpresent	3abr	Yes	1mrlab	No	No	Yes
K0119	Sundarijal	Ababsent	4abr	Yes	3mrlab	Yes	No	Yes
K0120	Sundarijal	Abpresent	1abr	Yes	1mrlab	No	No	No
K0121	Sundarijal	Abpresent	4abr	Yes	2mrlab	Yes	No	Yes
K0122	Sundarijal	Ababsent	0abr	No	0mrlab	No	No	No
K0123	Sundarijal	Abpresent	3abr	Yes	2mrlab	No	No	Yes
K0124	Sundarijal	Abpresent	3abr	Yes	2mrlab	Yes	No	No
K0125	Sundarijal	Ababsent	0abr	No	0mrlab	No	No	No
K0126	Sundarijal	Abpresent	4abr	Yes	1mrlab	Yes	No	Yes
K0127	Sundarijal	Abpresent	3abr	Yes	1mrlab	No	No	Yes
K0128	Sundarijal	Ababsent	2abr	No	0mrlab	Yes	No	Yes
K0129	Sundarijal	Ababsent	2abr	No	0mrlab	Yes	No	Yes
K0130	Sundarijal	Ababsent	1abr	No	0mrlab	Yes	No	No
K0131	Sundarijal	Ababsent	0abr	No	0mrlab	No	No	No
K0132	Sundarijal	Abpresent	3abr	Yes	2mrlab	Yes	No	Yes
K0133	Sundarijal	Abpresent	3abr	Yes	1mrlab	No	No	Yes
K0134	Sundarijal	Abpresent	3abr	Yes	2mrlab	No	No	Yes
K0135	Sundarijal	Abpresent	3abr	Yes	2mrlab	Yes	No	Yes
K0136	Sundarijal	Ababsent	2abr	No	0mrlab	Yes	No	No
K0137	Sundarijal	Abpresent	1abr	Yes	1mrlab	No	No	Yes
K0138	Sundarijal	Abpresent	2abr	Yes	1mrlab	Yes	No	No
K0139	Sundarijal	Abpresent	2abr	Yes	2mrlab	No	No	Yes
K0140	Sundarijal	Abpresent	3abr	Yes	2mrlab	Yes	No	Yes
K0141	Sundarijal	Ababsent	0abr	No	0mrlab	No	No	No
K0142	Sundarijal	Abpresent	2abr	Yes	1mrlab	Yes	No	Yes
K0143	Sundarijal	Abpresent	4abr	Yes	1mrlab	Yes	No	Yes
K0144	Sundarijal	Ababsent	0abr	No	0mrlab	No	No	No
K0145	Sundarijal	Ababsent	3abr	No	0mrlab	Yes	No	Yes

K0146	Sundarijal	Abpresent	2abr	Yes	2mrlab	No	No	No
K0147	Sundarijal	Ababsent	0abr	No	0mrlab	No	No	No
K0148	Sundarijal	Ababsent	1abr	No	0mrlab	No	No	Yes
K0149	Sundarijal	Ababsent	0abr	No	0mrlab	No	No	No
K0150	Sundarijal	Ababsent	0abr	No	0mrlab	No	No	No
K0151	Sundarijal	Abpresent	3abr	Yes	1mrlab	No	No	Yes
K0152	Sundarijal	Ababsent	0abr	No	0mrlab	No	No	No
K0153	Sundarijal	Abpresent	4abr	Yes	1mrlab	Yes	No	Yes
K0154	Sundarijal	Ababsent	0abr	No	0mrlab	No	No	No
K0201	Budhanika	Ababsent	1abr	No	0mrlab	No	No	No
K0202	Budhanika	Abpresent	2abr	Yes	2mrlab	No	No	Yes
K0203	Budhanika	Abpresent	3abr	Yes	1mrlab	No	No	Yes
K0204	Budhanika	Abpresent	4abr	Yes	1mrlab	Yes	No	Yes
K0205	Budhanika	Abpresent	2abr	Yes	1mrlab	No	No	No
K0206	Budhanika	Abpresent	4abr	Yes	2mrlab	Yes	No	Yes
K0207	Budhanika	Abpresent	4abr	Yes	2mrlab	Yes	No	Yes
K0208	Budhanika	Abpresent	3abr	Yes	1mrlab	No	No	Yes
K0209	Budhanika	Abpresent	3abr	Yes	1mrlab	Yes	Yes	No
K0210	Budhanika	Abpresent	1abr	Yes	1mrlab	Yes	Yes	No
K0301	Balaju	Ababsent	2abr	No	0mrlab	No	No	No
K0302	Balaju	Abpresent	1abr	Yes	1mrlab	No	No	Yes
K0303	Balaju	Abpresent	3abr	Yes	2mrlab	No	No	Yes
K0304	Balaju	Abpresent	3abr	Yes	2mrlab	No	No	Yes
K0305	Balaju	Ababsent	1abr	No	0mrlab	No	No	No
K0306	Balaju	Ababsent	1abr	No	0mrlab	No	No	Yes
K0307	Balaju	Abpresent	3abr	Yes	1mrlab	Yes	No	Yes
K0308	Balaju	Abpresent	4abr	Yes	2mrlab	Yes	No	Yes
K0309	Balaju	Ababsent	0abr	No	0mrlab	No	No	No
K0310	Balaju	Abpresent	2abr	Yes	1mrlab	No	No	No
K0311	Balaju	Abpresent	3abr	Yes	1mrlab	Yes	No	No
K0312	Balaju	Abpresent	3abr	Yes	1mrlab	Yes	No	No
K0313	Balaju	Abpresent	2abr	Yes	1mrlab	Yes	No	Yes
K0314	Balaju	Ababsent	1abr	No	0mrlab	No	No	No
K0315	Balaju	Ababsent	1abr	No	0mrlab	No	No	No
K0316	Balaju	Abpresent	4abr	Yes	1mrlab	Yes	No	Yes
K0317	Balaju	Ababsent	3abr	No	0mrlab	Yes	No	No
K0318	Balaju	Abpresent	3abr	Yes	2mrlab	No	No	Yes
K0319	Balaju	Abpresent	2abr	Yes	2mrlab	No	No	No
K0320	Balaju	Abpresent	3abr	Yes	1mrlab	Yes	Yes	No
K0321	Balaju	Abpresent	2abr	Yes	1mrlab	No	No	No
K0322	Balaju	Ababsent	1abr	No	0mrlab	Yes	No	No
K0323	Balaju	Ababsent	2abr	No	0mrlab	No	No	No
K0324	Balaju	Abpresent	2abr	Yes	1mrlab	No	No	No
K0325	Balaju	Ababsent	0abr	No	0mrlab	No	No	No
K0326	Balaju	Ababsent	0abr	No	0mrlab	No	No	No
K0401	Ramkot	Abpresent	3abr	Yes	2mrlab	No	No	Yes
K0402	Ramkot	Ababsent	2abr	No	0mrlab	No	No	Yes
K0403	Ramkot	Ababsent	1abr	No	0mrlab	No	No	Yes

K0404	Ramkot	Abpresent	2abr	Yes	1mrlab	No	No	No
K0405	Ramkot	Abpresent	4abr	Yes	2mrlab	Yes	No	Yes
K0406	Ramkot	Ababsent	2abr	No	0mrlab	Yes	No	Yes
K0407	Ramkot	Abpresent	2abr	Yes	1mrlab	No	No	No
K0408	Ramkot	Abpresent	4abr	Yes	1mrlab	Yes	No	Yes
K0501	Chandragir	Ababsent	3abr	No	0mrlab	Yes	No	Yes
K0502	Chandragir	Ababsent	0abr	No	0mrlab	No	No	No
K0503	Chandragir	Ababsent	0abr	No	0mrlab	No	No	No
K0504	Chandragir	Ababsent	0abr	No	0mrlab	No	No	No
K0505	Chandragir	Ababsent	4abr	No	0mrlab	Yes	No	Yes
K0506	Chandragir	Abpresent	2abr	Yes	1mrlab	Yes	Yes	No
K0507	Chandragir	Ababsent	3abr	No	0mrlab	Yes	No	No
K0508	Chandragir	Abpresent	2abr	Yes	1mrlab	No	No	Yes
K0509	Chandragir	Abpresent	3abr	Yes	1mrlab	Yes	No	Yes
K0510	Chandragir	Abpresent	2abr	Yes	1mrlab	No	No	Yes
K0511	Chandragir	Abpresent	2abr	Yes	1mrlab	Yes	No	Yes
K0601	Kirtipur	Ababsent	0abr	No	0mrlab	No	No	No
K0602	Kirtipur	Ababsent	0abr	No	0mrlab	No	No	No
K0603	Kirtipur	Abpresent	2abr	Yes	2mrlab	No	No	No
K0604	Kirtipur	Ababsent	0abr	No	0mrlab	No	No	No
K0605	Kirtipur	Abpresent	3abr	Yes	2mrlab	No	No	Yes
K0606	Kirtipur	Abpresent	3abr	Yes	3mrlab	Yes	Yes	No
K0607	Kirtipur	Abpresent	3abr	Yes	1mrlab	Yes	No	Yes
K0608	Kirtipur	Abpresent	2abr	Yes	1mrlab	No	No	No
K0609	Kirtipur	Abpresent	1abr	Yes	1mrlab	Yes	Yes	No
K0610	Kirtipur	Abpresent	4abr	Yes	1mrlab	Yes	No	Yes
K0611	Kirtipur	Abpresent	4abr	Yes	1mrlab	Yes	No	Yes
K0612	Kirtipur	Abpresent	3abr	Yes	3mrlab	Yes	Yes	No
K0613	Kirtipur	Abpresent	3abr	Yes	1mrlab	No	No	Yes
K0614	Kirtipur	Ababsent	2abr	No	0mrlab	Yes	No	No
K0615	Kirtipur	Abpresent	3abr	Yes	2mrlab	Yes	No	No
K0616	Kirtipur	Abpresent	4abr	Yes	2mrlab	Yes	No	Yes
K0617	Kirtipur	Ababsent	2abr	No	0mrlab	Yes	No	Yes
K0618	Kirtipur	Abpresent	3abr	Yes	1mrlab	Yes	No	No
K0619	Kirtipur	Abpresent	1abr	Yes	1mrlab	No	No	No
K0620	Kirtipur	Ababsent	2abr	No	0mrlab	No	No	No
K0621	Kirtipur	Abpresent	2abr	Yes	1mrlab	No	No	No
K0701	Godawari	Ababsent	0abr	No	0mrlab	No	No	No
K0702	Godawari	Ababsent	1abr	No	0mrlab	No	No	No
K0703	Godawari	Ababsent	0abr	No	0mrlab	No	No	No
K0704	Godawari	Ababsent	0abr	No	0mrlab	No	No	No
K0705	Godawari	Abpresent	4abr	Yes	2mrlab	Yes	No	Yes
K0706	Godawari	Ababsent	3abr	No	0mrlab	No	No	Yes
K0707	Godawari	Abpresent	2abr	Yes	2mrlab	No	No	No
K0708	Godawari	Ababsent	2abr	Yes	2mrlab	No	No	No
K0709	Godawari	Ababsent	3abr	No	0mrlab	Yes	No	Yes
K0710	Godawari	Abpresent	0abr	No	0mrlab	No	No	No

Ciprofloxacin Above MRL	Sulphamethazine presence	Sulphamethazine above MRL	Sulfamethoxacole presence	Sulfamethoxacole above MRL	Responsible Milking	Last AB Use	AB administer by staffs	AB administer by para/vets
No	Yes	Yes	Yes	Yes	Male	1month	No	Yes
No	Yes	No	Yes	Yes	Male	1month	No	Yes
No	Yes	Yes	Yes	Yes	Male	1year	Yes	Yes
No	Yes	No	Yes	No	Female	1month	Yes	Yes
No	Yes	Yes	Yes	Yes	Female	6months	Yes	Yes
No	No	No	No	No	Female	1year	No	Yes
No	Yes	No	No	No	Female	1month	No	No
No	Yes	No	Yes	Yes	Female	1month	Yes	Yes
No	Yes	No	No	No	Female	unknown	No	Yes
Yes	Yes	Yes	No	No	Male	1month	No	Yes
No	Yes	No	No	No	Male	6months	No	Yes
No	No	No	Yes	Yes	Male	6months	No	Yes
No	Yes	Yes	Yes	No	Female	1month	Yes	Yes
No	No	No	No	No	Female	1year	Yes	Yes
Yes	No	No	No	No	Female	6months	No	Yes
No	Yes	No	Yes	Yes	Female	1year	No	Yes
No	Yes	Yes	Yes	Yes	Male	6months	No	Yes
No	Yes	Yes	Yes	No	Female	6months	Yes	Yes
Yes	Yes	Yes	Yes	Yes	Female	6months	No	Yes
No	Yes	Yes	No	No	Female	1month	Yes	Yes
No	Yes	Yes	Yes	Yes	Female	1month	No	Yes
No	No	No	No	No	Female	1year	Yes	Yes
Yes	Yes	Yes	Yes	No	Female	unknown	Yes	Yes
No	Yes	Yes	Yes	Yes	Female	6months	No	No
No	No	No	No	No	Male	unknown	Yes	Yes
No	Yes	Yes	Yes	No	Female	unknown	Yes	Yes
No	Yes	Yes	Yes	No	Female	6months	No	No
No	No	No	No	No	Female	1year	Yes	No
No	No	No	No	No	Female	unknown	No	No
No	No	No	No	No	Female	1year	No	No
No	No	No	No	No	Female	6months	Yes	Yes
Yes	Yes	Yes	No	No	Female	1year	No	Yes
No	Yes	Yes	Yes	No	Male	unknown	Yes	Yes
No	Yes	Yes	Yes	Yes	Female	6months	Yes	Yes
Yes	Yes	Yes	No	No	Female	1year	No	Yes
No	Yes	No	No	No	Male	6months	No	No
Yes	No	No	No	No	Female	6months	Yes	Yes
No	No	No	Yes	Yes	Female	unknown	Yes	Yes
Yes	Yes	Yes	No	No	Male	6months	No	No
Yes	Yes	Yes	No	No	Female	6months	Yes	Yes
No	No	No	No	No	Male	6months	Yes	Yes
Yes	No	No	No	No	Male	1year	No	Yes
No	Yes	Yes	Yes	No	Male	unknown	No	Yes
No	No	No	No	No	Male	unknown	No	Yes

No	Yes	No	No	No	Male	unknown	Yes	Yes
No	Yes	Yes	Yes	Yes	Female	1year	Yes	Yes
No	No	No	No	No	Female	6months	Yes	Yes
No	No	No	No	No	Female	1month	Yes	Yes
No	No	No	No	No	Female	6months	No	No
No	No	No	No	No	Male	unknown	Yes	Yes
No	Yes	Yes	Yes	No	Male	6months	Yes	Yes
No	No	No	No	No	Male	6months	Yes	Yes
No	Yes	Yes	Yes	No	Female	6months	Yes	Yes
No	No	No	No	No	Male	6months	Yes	Yes
No	No	No	Yes	No	Female	unknown	No	Yes
Yes	Yes	Yes	No	No	Female	unknown	No	Yes
Yes	Yes	No	Yes	No	Female	unknown	No	Yes
Yes	Yes	No	Yes	No	Female	1year	No	Yes
No	Yes	No	Yes	Yes	Male	unknown	Yes	No
Yes	Yes	Yes	Yes	No	Female	unknown	Yes	Yes
Yes	Yes	Yes	Yes	No	Female	unknown	No	No
Yes	Yes	No	Yes	No	Female	unknown	No	Yes
No	Yes	No	Yes	No	Male	6months	No	No
No	No	No	No	No	Female	1month	No	Yes
No	Yes	No	Yes	No	Female	1month	Yes	Yes
Yes	No	No	No	No	Female	1month	Yes	Yes
Yes	Yes	Yes	Yes	No	Male	6months	Yes	Yes
Yes	Yes	Yes	Yes	No	Male	1year	Yes	Yes
No	Yes	No	No	No	Male	1month	Yes	Yes
No	No	No	No	No	Female	unknown	No	Yes
No	Yes	Yes	No	No	Female	1month	No	No
No	Yes	Yes	Yes	Yes	Female	6months	Yes	Yes
No	No	No	No	No	Female	1year	No	No
No	Yes	No	Yes	Yes	Female	1month	Yes	Yes
No	Yes	Yes	Yes	No	Female	1year	Yes	Yes
No	Yes	Yes	Yes	No	Female	1year	Yes	Yes
Yes	No	No	No	No	Female	unknown	Yes	Yes
No	Yes	No	No	No	Female	1year	Yes	Yes
No	No	No	Yes	No	Female	unknown	Yes	Yes
No	Yes	Yes	Yes	No	Male	6months	Yes	Yes
No	Yes	No	Yes	No	Female	6months	Yes	Yes
Yes	Yes	Yes	Yes	No	Male	6months	No	No
No	Yes	Yes	Yes	Yes	Male	1month	Yes	Yes
No	Yes	No	Yes	No	Female	6months	Yes	Yes
No	Yes	No	Yes	Yes	Male	1month	Yes	Yes
No	No	No	No	No	Female	1year	Yes	Yes
No	Yes	No	Yes	No	Female	unknown	No	Yes
No	Yes	No	Yes	Yes	Female	1year	Yes	Yes
No	No	No	No	No	Female	1year	Yes	Yes
No	No	No	No	No	Female	6months	Yes	Yes

No	Yes	Yes	Yes	Yes	Male	6months	Yes	No
No	No	No	Yes	No	Female	1month	Yes	Yes
No	No	No	No	No	Female	unknown	Yes	Yes
No	Yes	Yes	Yes	No	Female	6months	No	Yes
Yes	Yes	Yes	Yes	No	Male	1year	Yes	Yes
No	No	No	No	No	Male	6months	No	Yes
No	Yes	Yes	Yes	No	Female	6months	Yes	No
No	Yes	Yes	Yes	No	Male	6months	Yes	No
No	Yes	No	No	No	Male	6months	Yes	No
No	No	No	No	No	Female	6months	Yes	Yes
No	No	No	No	No	Female	6months	Yes	Yes
No	No	No	No	No	Female	6months	Yes	Yes
No	Yes	No	Yes	No	Female	6months	No	Yes
No	No	No	Yes	No	3	unknown	Yes	No
No	Yes	No	Yes	No	Female	6months	No	Yes
No	Yes	Yes	No	No	Female	unknown	Yes	Yes
Yes	Yes	No	No	No	Male	1year	No	No
Yes	Yes	No	No	No	Male	1month	Yes	Yes
Yes	No	No	No	No	Male	1month	No	No
No	No	No	No	No	Female	6months	Yes	No
No	No	No	No	No	Female	1year	Yes	Yes
No	Yes	Yes	Yes	Yes	Male	1month	Yes	Yes
No	No	No	No	No	Female	unknown	Yes	No
Yes	Yes	Yes	Yes	No	Female	1month	No	No
No	Yes	Yes	Yes	Yes	Female	6months	No	Yes
No	Yes	Yes	No	No	Female	1year	No	Yes
No	Yes	No	Yes	Yes	Female	1month	Yes	Yes
No	No	No	No	No	Female	unknown	No	Yes
No	Yes	Yes	Yes	No	Female	unknown	No	Yes
No	Yes	Yes	Yes	No	Female	1month	No	Yes
No	Yes	Yes	Yes	Yes	Male	6months	Yes	Yes
No	Yes	No	Yes	Yes	Female	unknown	Yes	Yes
No	Yes	No	No	No	Female	6months	No	No
No	Yes	Yes	Yes	Yes	Female	6months	Yes	Yes
Yes	Yes	Yes	Yes	No	Female	6months	Yes	Yes
No	No	No	No	No	Female	6months	Yes	Yes
No	Yes	No	Yes	Yes	Female	unknown	No	Yes
No	Yes	Yes	No	No	Female	1month	No	Yes
No	Yes	No	Yes	No	Female	6months	No	Yes
No	Yes	Yes	Yes	No	Female	unknown	Yes	Yes
No	No	No	No	No	Female	6months	Yes	Yes
No	Yes	No	No	No	Female	6months	Yes	Yes
No	No	No	No	No	Male	1year	Yes	Yes
No	No	No	No	No	Female	6months	Yes	Yes
No	Yes	Yes	Yes	Yes	Male	unknown	Yes	Yes
No	Yes	No	Yes	No	Female	6months	Yes	Yes
No	Yes	Yes	Yes	Yes	Female	6months	No	Yes
No	Yes	Yes	Yes	Yes	Female	1year	Yes	Yes

No	Yes	No	No	No	Female	unknown	Yes	Yes
No	No	No	No	No	Female	1month	No	Yes

Visit chemists	Visit Vets	Vets Distance	Frequency visit	Vitamin/ supplement use	Freq Selling Milk	Desinfecting Sheds	Cleaning Animals	Withholding Milk
Yes	Yes	1to5KM	5to10	Yes	Twice daily	Weekly	Monthly	No
Yes	Yes	1to5KM	5to10	Yes	Twice daily	Weekly	Monthly	No
No	Yes	1to5KM	less5	Yes	Twice daily	Weekly	Monthly	Yes
Yes	Yes	less1KM	less5	Yes	Twice daily	Monthly	Monthly	Yes
Yes	Yes	1to5KM	5to10	Yes	Twice daily	Weekly	Monthly	No
Yes	Yes	1to5KM	5to10	Yes	Twice daily	Monthly	Monthly	No
Yes	Yes	1to5KM	5to10	Yes	Twice daily	Weekly	Weekly	No
Yes	Yes	1to5KM	more10	Yes	Twice daily	Monthly	Weekly	Yes
Yes	Yes	1to5KM	less5	Yes	Twice daily	Monthly	Monthly	Yes
Yes	Yes	1to5KM	5to10	Yes	Twice daily	Monthly	Monthly	Yes
Yes	Yes	1to5KM	5to10	Yes	Twice daily	Weekly	Weekly	Yes
Yes	Yes	1to5KM	less5	Yes	Twice daily	Weekly	Weekly	Yes
Yes	Yes	1to5KM	less5	Yes	Twice daily	Monthly	Monthly	No
Yes	Yes	more5KM	5to10	Yes	Twice daily	Weekly	Weekly	Yes
Yes	Yes	less1KM	5to10	Yes	Twice daily	Monthly	Weekly	Yes
Yes	Yes	1to5KM	less5	Yes	Twice daily	Monthly	Monthly	No
Yes	Yes	1to5KM	less5	Yes	Twice daily	Weekly	Weekly	No
Yes	Yes	1to5KM	less5	Yes	Twice daily	Monthly	Monthly	Yes
Yes	Yes	1to5KM	less5	Yes	Twice daily	Monthly	Monthly	No
Yes	Yes	1to5KM	less5	Yes	Twice daily	Weekly	Monthly	No
Yes	Yes	1to5KM	5to10	Yes	Twice daily	Monthly	Monthly	No
Yes	Yes	1to5KM	less5	Yes	Twice daily	Monthly	Monthly	No
Yes	Yes	1to5KM	less5	No	Twice daily	Weekly	Monthly	No
Yes	Yes	1to5KM	less5	Yes	Twice daily	Weekly	Weekly	No
Yes	Yes	1to5KM	less5	Yes	Twice daily	Weekly	Monthly	No
Yes	Yes	1to5KM	5to10	Yes	Twice daily	Monthly	Monthly	No
Yes	Yes	1to5KM	5to10	Yes	Twice daily	Monthly	Monthly	No
Yes	Yes	1to5KM	less5	Yes	Twice daily	Weekly	Monthly	No
Yes	Yes	1to5KM	5to10	No	Twice daily	Monthly	Monthly	No
Yes	Yes	1to5KM	5to10	Yes	Twice daily	Monthly	Monthly	No
Yes	Yes	1to5KM	less5	Yes	Twice daily	Monthly	Monthly	No
Yes	Yes	1to5KM	5to10	Yes	Twice daily	Monthly	Monthly	No
Yes	Yes	1to5KM	less5	Yes	Twice daily	Monthly	Monthly	No
Yes	Yes	1to5KM	less5	Yes	Twice daily	Monthly	Monthly	Yes
Yes	Yes	1to5KM	5to10	Yes	Twice daily	Weekly	Weekly	Yes
Yes	Yes	1to5KM	5to10	Yes	Twice daily	Weekly	Monthly	Yes
Yes	Yes	more5KM	5to10	Yes	Twice daily	Weekly	Monthly	No
Yes	Yes	1to5KM	5to10	Yes	Twice daily	Monthly	Monthly	No
Yes	Yes	1to5KM	less5	Yes	Twice daily	Monthly	Monthly	No
Yes	Yes	more5KM	5to10	Yes	Twice daily	Weekly	Monthly	No
Yes	Yes	more5KM	5to10	Yes	Twice daily	Monthly	Weekly	No
Yes	Yes	1to5KM	less5	Yes	Twice daily	Monthly	Monthly	No
Yes	Yes	more5KM	5to10	Yes	Twice daily	Monthly	Monthly	No
Yes	Yes	more5KM	less5	No	Twice daily	Monthly	Monthly	No

Yes	Yes	more5KM	5to10	Yes	Twice daily	Monthly	Monthly	No
Yes	Yes	1to5KM	5to10	Yes	Twice daily	Weekly	Monthly	No
Yes	Yes	1to5KM	5to10	Yes	Twice daily	Weekly	Weekly	No
Yes	Yes	more5KM	5to10	Yes	Twice daily	Weekly	Weekly	No
Yes	Yes	more5KM	5to10	Yes	Twice daily	Weekly	Weekly	Yes
Yes	Yes	more5KM	5to10	Yes	Twice daily	Monthly	Monthly	Yes
Yes	Yes	more5KM	less5	Yes	Twice daily	Weekly	Monthly	No
Yes	Yes	more5KM	less5	Yes	Twice daily	Monthly	Weekly	Yes
Yes	Yes	more5KM	5to10	Yes	Twice daily	Weekly	Weekly	No
Yes	Yes	more5KM	less5	Yes	Twice daily	Weekly	Weekly	Yes
Yes	Yes	1to5KM	less5	Yes	Twice daily	Never	Weekly	Yes
Yes	Yes	1to5KM	less5	Yes	Twice daily	Never	Daily	No
Yes	Yes	1to5KM	less5	Yes	Twice daily	Monthly	Daily	Yes
Yes	Yes	1to5KM	less5	Yes	Twice daily	Never	Never	Yes
Yes	No	Noinfo	less5	Yes	Twice daily	Never	Daily	Yes
Yes	Yes	1to5KM	less5	Yes	Twice daily	Weekly	Daily	No
Yes	Yes	more5KM	less5	Yes	Twice daily	Never	Weekly	No
Yes	Yes	1to5KM	less5	Yes	Twice daily	Weekly	Weekly	No
Yes	Yes	1to5KM	5to10	Yes	Twice daily	Monthly	Weekly	Yes
Yes	Yes	1to5KM	5to10	Yes	Twice daily	Daily	Daily	No
Yes	Yes	1to5KM	less5	No	Twice daily	Monthly	Monthly	Yes
Yes	Yes	1to5KM	5to10	Yes	Twice daily	Monthly	Monthly	Yes
Yes	Yes	1to5KM	less5	Yes	Twice daily	Weekly	Monthly	Yes
Yes	Yes	more5KM	5to10	No	Twice daily	Monthly	Monthly	Yes
Yes	Yes	1to5KM	less5	No	Twice daily	Monthly	Monthly	Yes
Yes	Yes	more5KM	5to10	Yes	Twice daily	Monthly	Monthly	Yes
Yes	Yes	more5KM	less5	Yes	Once daily	Monthly	Monthly	No
No	Yes	1to5KM	5to10	Yes	Once daily	Monthly	Monthly	No
Yes	Yes	more5KM	5to10	Yes	Once daily	Monthly	Monthly	Yes
Yes	Yes	more5KM	5to10	Yes	Twice daily	Monthly	Monthly	No
Yes	Yes	more5KM	less5	Yes	Twice daily	Monthly	Monthly	Yes
Yes	Yes	more5KM	5to10	Yes	Twice daily	Monthly	Weekly	No
Yes	Yes	more5KM	less5	Yes	Twice daily	Monthly	Weekly	No
Yes	Yes	more5KM	5to10	Yes	Twice daily	Monthly	Monthly	No
Yes	Yes	more5KM	5to10	No	Twice daily	Monthly	Monthly	No
Yes	Yes	1to5KM	5to10	No	Once daily	Monthly	Monthly	No
Yes	Yes	more5KM	5to10	Yes	Twice daily	Monthly	Monthly	Yes
Yes	Yes	more5KM	less5	No	Once daily	Monthly	Monthly	No
Yes	Yes	more5KM	less5	No	Twice daily	Never	Weekly	Yes
Yes	Yes	1to5KM	less5	No	Twice daily	Monthly	Monthly	No
Yes	Yes	1to5KM	less5	No	Twice daily	Monthly	Weekly	Yes
Yes	Yes	1to5KM	less5	No	Twice daily	Monthly	Monthly	No
Yes	Yes	more5KM	less5	Yes	Once daily	Monthly	Daily	No
Yes	Yes	more5KM	5to10	Yes	Twice daily	Weekly	Monthly	No
Yes	Yes	more5KM	less5	Yes	Once daily	Monthly	Monthly	No
Yes	Yes	more5KM	5to10	Yes	Once daily	Monthly	Monthly	Yes

Yes	Yes	more5KM	less5	Yes	Twice daily	Daily	Daily		Yes
Yes	Yes	1to5KM	5to10	Yes	Twice daily	Never	Weekly		Yes
Yes	Yes	1to5KM	more10	Yes	Twice daily	Monthly	Monthly		Yes
Yes	Yes	1to5KM	less5	Yes	Twice daily	Monthly	Weekly	No	
Yes	Yes	1to5KM	5to10	Yes	Twice daily	Weekly	Monthly	Yes	
Yes	Yes	1to5KM	5to10	Yes	Twice daily	Daily	Daily	Yes	
Yes	Yes	1to5KM	more10	No	Twice daily	Monthly	Daily	No	
Yes	Yes	more5KM	5to10	Yes	Twice daily	Monthly	Daily	Yes	
Yes	Yes	1to5KM	5to10	Yes	Twice daily	Monthly	Monthly	Yes	
Yes	Yes	more5KM	less5	No	Twice daily	Never	Never	No	
Yes	Yes	more5KM	less5	No	Once daily	Never	Never	No	
Yes	Yes	more5KM	less5	No	Once daily	Never	Monthly	No	
Yes	Yes	more5KM	5to10	Yes	Once daily	Monthly	Weekly	Yes	
Yes	Yes	more5KM	less5	No	Once daily	Never	Daily	Yes	
Yes	Yes	more5KM	less5	Yes	Once daily	Never	Monthly	No	
Yes	Yes	1to5KM	5to10	No	Once daily	Monthly	Daily	Yes	
Yes	Yes	1to5KM	less5	Yes	Twice daily	Monthly	Monthly	No	
Yes	Yes	1to5KM	less5	Yes	Twice daily	Never	Monthly	Yes	
Yes	Yes	1to5KM	5to10	Yes	Twice daily	Weekly	Monthly	No	
Yes	Yes	less1KM	5to10	Yes	Twice daily	Weekly	Monthly	No	
Yes	Yes	1to5KM	less5	Yes	Twice daily	Monthly	Monthly	No	
No	Yes	1to5KM	5to10	Yes	Twice daily	Weekly	Monthly	No	
No	No	less1KM	less5	Yes	Twice daily	Monthly	Monthly	No	
No	Yes	1to5KM	less5	Yes	Twice daily	Monthly	Monthly	No	
Yes	Yes	1to5KM	more10	Yes	Twice daily	Monthly	Daily	Yes	
Yes	Yes	1to5KM	5to10	Yes	Twice daily	Weekly	Monthly	Yes	
Yes	Yes	1to5KM	5to10	Yes	Twice daily	Weekly	Monthly	Yes	
Yes	No	more5KM	less5	Yes	Twice daily	Never	Monthly	Yes	
Yes	Yes	1to5KM	more10	Yes	Twice daily	Never	Monthly	No	
No	Yes	1to5KM	less5	Yes	Twice daily	Weekly	Weekly	Yes	
No	Yes	1to5KM	less5	Yes	Twice daily	Weekly	Monthly	No	
Yes	Yes	1to5KM	5to10	Yes	Twice daily	Monthly	Monthly	No	
No	Yes	1to5KM	5to10	Yes	Twice daily	Weekly	Weekly	No	
No	No	Noinfo	less5	Yes	Twice daily	Monthly	Monthly	No	
Yes	Yes	1to5KM	5to10	Yes	Twice daily	Weekly	Monthly	No	
Yes	Yes	1to5KM	less5	Yes	Twice daily	Never	Monthly	No	
Yes	Yes	1to5KM	less5	Yes	Twice daily	Weekly	Monthly	No	
Yes	Yes	1to5KM	less5	Yes	Twice daily	Never	Daily	No	
Yes	Yes	1to5KM	5to10	Yes	Twice daily	Weekly	Monthly	Yes	
Yes	Yes	1to5KM	5to10	Yes	Twice daily	Weekly	Monthly	Yes	
Yes	Yes	1to5KM	more10	Yes	Twice daily	Weekly	Weekly	No	
Yes	Yes	1to5KM	5to10	Yes	Twice daily	Weekly	Monthly	Yes	
Yes	Yes	1to5KM	5to10	Yes	Twice daily	Monthly	Monthly	Yes	
Yes	Yes	1to5KM	5to10	Yes	Twice daily	Weekly	Monthly	No	
Yes	Yes	1to5KM	5to10	Yes	Twice daily	Never	Monthly	Yes	
Yes	Yes	1to5KM	less5	Yes	Twice daily	Never	Monthly	No	
Yes	Yes	1to5KM	5to10	Yes	Twice daily	Never	Monthly	Yes	
Yes	Yes	1to5KM	5to10	Yes	Twice daily	Monthly	Monthly	Yes	

Yes	Yes	1to5KM	less5	Yes	Twice daily	Monthly	Monthly	No
Yes	Yes	1to5KM	less5	Yes	Twice daily	Never	Weekly	No
